# Whole-exome sequencing identified a novel heterozygous mutation of *SALL1* and a new homozygous mutation of *PTPRQ* in a Chinese family with Townes-Brocks syndrome and hearing loss

**DOI:** 10.1186/s12920-021-00871-9

**Published:** 2021-01-21

**Authors:** Guangxian Yang, Yi Yin, Zhiping Tan, Jian Liu, Xicheng Deng, Yifeng Yang

**Affiliations:** 1 Department of Cardiothoracic Surgery, Hunan Children’s Hospital, No. 86 Ziyuan Road, Changsha, Hunan Province 410007 China; 2 Department of Cardiovascular Surgery, The Second Xiangya Hospital of Central South University, Changsha, China

**Keywords:** Townes-brocks syndrome, Hearing loss, SALL1 mutation, PTPRQ mutation

## Abstract

**Background:**

Previous studies have revealed that mutations of *Spalt Like Transcription Factor 1* (*SALL1*) are responsible for Townes-Brocks syndrome (TBS), a rare genetic disorder that is characterized by an imperforate anus, dysplastic ears, thumb malformations and other abnormalities, such as hearing loss, foot malformations, renal impairment with or without renal malformations, genitourinary malformations, and congenital heart disease. In addition, the *protein tyrosine phosphatase receptor type Q* (*PTPRQ*) gene has been identified in nonsyndromic hearing loss patients with autosomal recessive or autosomal dominant inherited patterns.

**Methods:**

A Chinese family with TBS and hearing loss was enrolled in this study. The proband was a two-month-old girl who suffered from congenital anal atresia with rectal perineal fistula, ventricular septal defect, patent ductus arteriosus, pulmonary hypertension (PH), and finger deformities. The proband’s father also had external ear deformity with deafness, toe deformities and PH, although his anus was normal. Further investigation found that the proband’s mother presented nonsyndromic hearing loss, and the proband’s mother’s parents were consanguine married. Whole-exome sequencing and Sanger sequencing were applied to detect the genetic lesions of TBS and nonsyndromic hearing loss.

**Results:**

Via whole-exome sequencing and Sanger sequencing of the proband and her mother, we identified a novel heterozygous mutation (ENST00000251020: c.1428_1429insT, p. K478QfsX38) of *SALL1* in the proband and her father who presented TBS phenotypes, and we also detected a new homozygous mutation [ENST00000266688: c.1057_1057delC, p. L353SfsX8)] of *PTPRQ* in the proband’s mother and uncle, who suffered from nonsyndromic hearing loss. Both mutations were located in the conserved sites of the respective protein and were predicted to be deleterious by informatics analysis.

**Conclusions:**

This study confirmed the diagnosis of TBS at the molecular level and expanded the spectrum of *SALL1* mutations and *PTPRQ* mutations. Our study may contribute to the clinical management and genetic counselling of TBS and hearing loss.

## Background

Townes-Brocks syndrome (TBS, OMIM: #107480) is a congenital genetic disorder characterized by the triad of atresia of the anus, dysplasia of the external ears and thumb deformity [1, 2]. Secondary features of the syndrome include hearing loss, foot deformity, renal insufficiency with or without renal malformations, urogenital malformations, and congenital heart disease (CHD) [3, 4]. TBS was first reported in 1972 by Townes and Brocks, who described a family with congenital anorectal malformations (ARMs), polydactyly of the thumb, and other skeletal abnormalities (metatarsal fusion, missing thumb bones, and multifinger deformity) [5]. At present, patients who are diagnosed with TBS should have at least two of the following three major features: an imperforate anus (84% of the cases), abnormally shaped ears (87% of the cases) and thumb malformations (89% of the cases) [6]

In 1998, Kohlhase et al. first identified two different heterozygous mutations of *Spalt Like Transcription Factor 1* (*SALL1*) in a family with three cases of TBS in two generations and in an unrelated family with a sporadic case of TBS [7]. Since then, more than ninety mutations of *SALL1* have been detected in TBS patients, and most were nonsense mutations and frameshift mutations. SALL1, a strong transcriptional repressor, is regulated by the sonic hedgehog pathway, which plays a crucial role in the development of multiple organs [8, 9]. In addition, some authors proposed the involvement of SALL1 in the regulation of higher-order chromatin structures and hypothesized that the protein may be a component of a distinct heterochromatin-dependent silencing process [10, 11]. Recently, studies have found that SALL1 can interact with ciliogenesis suppressors CCP110 and CEP97 and mutations in *SALL1* may disrupt the formation and function of cilia [1, 12].

Hearing loss or deafness (prevalence rate of 0.1%) is the most common sensory disorder and affects millions worldwide [13]. In terms of physical function and social movement, the quality of life of hearing loss patients is poor. Fifty percent of these cases are estimated to be caused by genetic factors. Previous studies have shown that mutations in *protein tyrosine phosphatase receptor type Q* (*PTPRQ*) may lead to autosomal recessive nonsyndromic hearing loss [14]. More than thirty mutations of *PTPRQ* have been identified in patients with hearing loss. Meanwhile, a recent study also found that the heterozygous c.6881G > A transition (NM_001145026.1) in the last coding exon of *PTPRQ* may lead to autosomal dominant nonsyndromic hearing loss [15, 16].

In this study, we identified a novel heterozygous mutation (ENST00000251020: c.1428_1429insT, p. K478QfsX38) of *SALL1* and a new homozygous mutation (ENST00000266688: c.1057_1057delC, p. L353SfsX8) of *PTPRQ* in a Chinese family that included two TBS patients and another two individuals with hearing loss by whole-exome sequencing.

## Methods

### Subjects

**Fig. 1:**
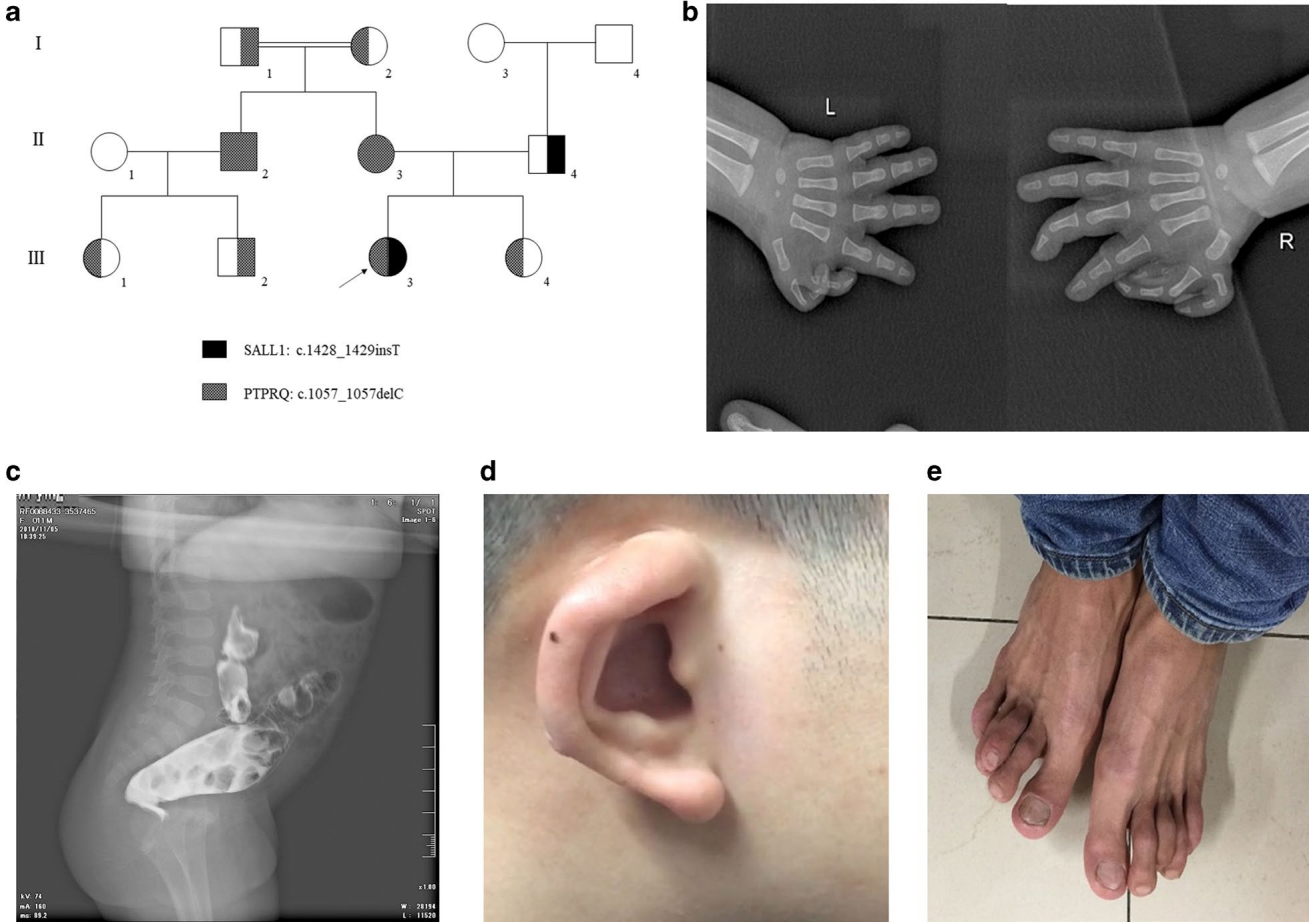
Clinical data for the family. **a** Pedigree of this family. Square indicates male, circle indicates female, and arrow indicates the proband. **b** Proband’s X-ray with anorectal deformity and fistula. **c** X-rays of the proband’s hands with a multifinger phenotype. **d** External ear deformity of the proband’s father. **e** Toe deformities of the proband’s father

The family, including ten persons, was investigated in this study (Fig. 1a). Peripheral blood samples of all these family members were collected and applied to isolate genomic DNA by a Universal Genomic DNA Extraction Kit (Solarbio, D2100) as we previously described [17]. In addition, 200 unrelated local healthy people were also enrolled to serve as normal controls. This study was approved by the Ethics Committee of Hunan Children’s Hospital, Changsha, China, and performed in accordance with the principles enshrined in the Declaration of Helsinki. The patients/participants provided their written informed consent to participate in this study.

### Whole-exome sequencing

We selected the proband (III-3) and her mother (II-3) to perform whole-exome sequencing. Exome capture and next-generation sequencing were conducted by the Novogene Bioinformatics Institute (Beijing, China). One microgram of qualified genomic DNA from each person was captured by the Agilent SureSelect Human All Exon kit V5 (Agilent Technologies, Inc., Santa Clara, USA) and sequenced using an Illumina HiSeq 4000 (Illumina Inc., San Diego, USA) [17]. Briefly, genomic DNA was randomly carved by a Covaris S220 sonicator (Covaris, Inc., Woburn, USA). Then, the fragmented DNAs underwent three enzymatic steps: end repair, A-tailing and adapter ligation. The adapter-ligated DNA fragments were amplified with Herculase II Fusion DNA Polymerase (Agilent). Finally, the exomes in the precapture libraries were captured by a SureSelect capture library kit (Agilent) [17]. After DNA quality assessment, the captured DNA library was used for next-generation sequencing on the Illumina HiSeq 4000 platform. After demultiplexing, we used Burrows Wheeler alignment to map the paired-end sequences to the human genome (NCBI Build 37/hg19). Downstream processing was carried out using the Genome Analysis Toolkit (GATK) as well as Varscan2 and Picard, and variant calls were performed with the GATK Haplotype Caller. Variant annotation referred to Ensemble release 82, and filtering was conducted by ANNOVAR Documentation [18].

Nonsynonymous SNPs or frameshift-causing INDELs with an alternative allele frequency > 0.005 in the NHLBI Exome Sequencing Project Exome Variant Server (ESP6500), dbSNP147 (http://www.ncbi.nlm.nih.gov/projects/SNP/index.html), the 1000 Genomes project (http://www.1000genomes.org/), and the ExAC database or in-house exome databases of Novogene (2500 exomes) were kicked before further analysis. Then, the SNVs and INDELs predicted by SIFT (http://sift.jcvi.org/), Polyphen2 (http://genetics.bwh.harvard.edu/pph2/) and MutationTaster (http://www.mutationtaster.org/) to be damaging remained were filtered. Finally, mutations were found in two affected members (I-2 and III-4) but were absent in the healthy individual (II-2) [18].

### Mutation validation and cosegregation analysis

After the filtering process, all mutations found for this family were validated by Sanger sequencing. The primer sequences were as follows: SALL1: forward, 5′-GGTACACATGGGCACTCACA-3′; reverse, 5′-GCCACCATAGGTCGCATTCT-3′, and PTPRQ: forward, 5′-TGTTGTCTTTGGCTCTGTACTT-3′; reverse, 5′-GTTCTTACCATCTGGTGGAGTG-3′. The sequences of the polymerase chain reaction (PCR) products were determined using the ABI 3100 Genetic Analyser (ABI, Foster City, CA, USA) [19].

## Results

### Clinical data

The proband (III-3), a two-month-old girl, was from the mountainous area of southern China and suffered from congenital anal atresia with rectal perineal fistula (Fig. 1b), ventricular septal defect, patent ductus arteriosus, pulmonary hypertension (PS), and finger deformities (Fig. 1c). A family history survey found that the proband’s father (II-4) also suffered from external ear deformity with deafness (Fig. 1d), toe deformities (Fig. 1e) and PS, although his anus was normal. Both the proband (III-3) and her father (II-4) were suspected of having TBS.

During the family history investigation, we found another interesting truth: both the proband’s mother (II-3) and uncle (II-2) were isolated deafness patients and their parents (I-1 and I-2) were consanguine married. No other deafness patients were detected. The proband (III-3) underwent surgical treatment of CHD as well as anoplasty surgery for ARM after three months.

### Genetic analysis

**Fig. 2:**
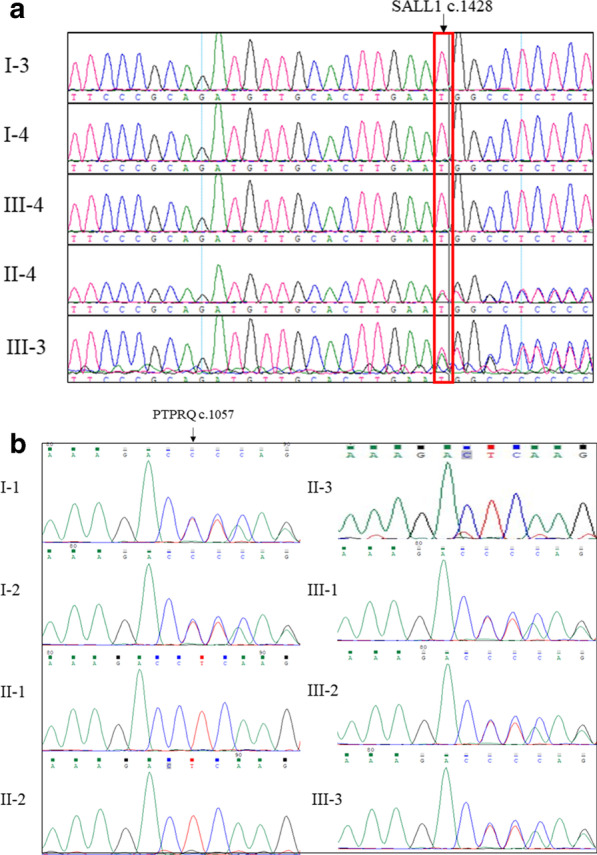
Genetic analysis of the family. **a** Sanger DNA sequencing chromatogram demonstrates the heterozygosity for a *SALL1* mutation (c.1428_1429insT, p. K478QfsX38) in the proband and her father. **b** Sanger DNA sequencing chromatogram demonstrates the homozygosity for a *PTPRQ* mutation (c.1057_1057delC, p. L353SfsX8) in the proband’ mother and uncle

Different genetic lesions may underlie a family with hearing loss and congenital malformations. The proband (III-3) (Additional file 1) and her mother (II-3) (Additional file 2) to perform whole-exome sequencing analysis. After data screening, we detected a novel heterozygous mutation (ENST00000251020: c.1428_1429insT, p. K478QfsX38) of *SALL1* in the proband (III-3) (Fig. 2a) and a new homozygous mutation (ENST00000266688: c.1057_1057delC, p. L353SfsX8) of *PTPRQ* in the proband’s mother (II-3) (Fig. 2b). No other meaningful mutations were identified. Further cosegregation analysis found that the proband’s father (II-4) carried the heterozygous mutation of *SALL1* (Fig. 2a) and both the proband (III-3) and her father (II-4) were confirmed to have TBS. Sanger sequencing further found that the proband’s uncle (II-2) carried a homozygous mutation in *PTPRQ* while the proband’s grandfather (I-1), grandmother (I-2) and proband (III-3) carried a heterozygous mutation (ENST00000266688: c.1057_1057delC, p. L353SfsX8) of *PTPRQ* (Fig. 2b). Both novel mutations (SALL1: c.1428_1429insT and PTPRQ: c.1057_1057delC) were located in the conserved sites of the respective protein and predicted to be deleterious by informatics analysis. Both mutations were also absent in our 200 local control cohorts as well as other public databases, such as ExAC (http://exac.broadinstitute.org) and genomAD (https://gnomad.broadinstitute.org). Moreover, ACMG guidelines [20] indicate that both mutations are pathogenic.

## Discussion

### SALL1

The current research confirms that TBS syndrome is an autosomal dominant genetic disease. The heterozygous mutation of the zinc finger protein transcription regulation gene *SALL1* at 16q12.1 is the molecular basis of the disease. In 2005, Botzenhart et al. reported that most of the *SALL1* gene mutations occurred in or within the 5-prime encoding the first double zinc finger region among the 35 identified genetic mutations [21]. The *SALL1* gene contains three determined exons, with 11 WT1-binding sites and 1 SIX1-binding site. The 5 prime number flanking regions do not contain TATA or CAAT boxes, although they are rich in GC and contain more GC boxes [22]. In addition to the *SALL1* gene, recent studies found that mutations in the *Dishevelled Binding Antagonist Of Beta Catenin 1* (*DACT1*) gene located on chromosome 14q23 may also lead to TBS, which is called Townes-Brocks syndrome-2 (TBS2; 617466) [23].

Some families with Townes-Brocks syndrome have shown increased clinical severity over generations, thus presenting genetic anticipation. Sudo et al. reported an antedating Japanese family in which a four-year-old female proband was heterozygous for a frameshift mutation in the *SALL1* gene, the first generation of affected patients (the proband’s grandfather and great aunt) showed polydactyly and deafness, the second generation (the proband’s mother and uncle) had kidney and anal abnormalities, and the third generation (the proband) showed the most serious symptoms of anteriorly placed and stenosed anus and ventricular septal defects (VSDs) [2]. Researchers noted that the severity of the phenotype with each succeeding generation was an anticipation-like increase in this family and showed that similar increases in clinical severity had been observed in other TBS families, frequently in patients who inherited the disease from their mothers.

In our study, we identified a novel heterozygous mutation (ENST00000251020: c.1428_1429insT, p. K478QfsX38) of *SALL1* in a two-generation family with TBS. The first generation (II-4) only showed toe deformities, deafness, external ear deformities and PS, although more serious complications, including multiple CHDs and ARMs, were present in the second generation (III-3). At present, this proband (III-3) has no sensorineural deafness and only shows mild conductive deafness, which is related to otitis media that was present during the examination. Moreover, the child may not show sensorineural deafness; thus, follow-up review is still needed in the future.

### PTPRQ

PTPRQ plays an important role in inner ear development and hair cell differentiation and maturation [24, 25] and is necessary for the normal maturation of shaft connectors and developing hair bundles in the mammalian cochlea. There are four PTPRQ isotypes, and each isotype is composed of a transmembrane domain, fibronectin type 3 domain and phosphatase domain [26]. Previous studies suggested that PTPRQ and myosin VI can form a complex, which may dynamically maintain the organization coated on the surface of the stereocilia base cells and may help maintain the overall structure of the stereocilia bundles [27]. PTPRQ knockout mice have significantly longer hair strands and suffer from the loss and fusion of hair cell static cilia. Researchers have also observed a simultaneous loss of function of the vestibular system in these mice [24]. Vestibular evoked potentials do not occur in most *PTPRQ* gene knockout mice, and subtle but obvious defects of evoked potentials are detected in mutant mice [24]. Goodyear et al. pointed out that the lack of PTPRQ in mice causes the apical membrane of hair cells to separate from the potential actin cytoskeleton and leads to the fusion of stereocilia [28]. PTPRQ mutations may alter the morphology and stereocilia and further lead to the gradual loss of cochlear hair cells and subsequent deafness [28].

Previous studies have proven that homozygous mutation of *PTPRQ* may lead to nonsyndromic hearing loss. In 2010, Arg457Gly (currently Arg239Gly) and Tyr497X (currently Tyr279X) were first identified by Schraders et al. in deaf families from Morocco and the Netherlands. Hashem et al. found that the cause of DFNB84 in Palestinian deaf families was Gln429X in *PTPRQ* [26]. In 2015, Qing Sang et al. identified c.1617insT (p. L8fsX18) and c.2714delA (p. E909fsX922) as new compound heterozygous mutations in the *PTPRQ* gene in a Kazakh family from Xinjiang, which was the first report of a *PTPRQ* gene mutation in China [29]. In the last coding exon (exon 45) of the *PTPRQ* gene, Eisenberger et al. identified a heterozygous c.6881G>A transition, which resulted in a p. W2294X substitution in affected members in four generations of German families in which the age at onset of hearing loss ranged from early childhood to the third decade, thus confirming that the *PTPRQ* gene was also a new autosomal dominant nonsyndromic hearing loss gene [16]**.**

In this study, we identified a new homozygous mutation (ENST00000266688: c.1057_1057delC, p. L353SfsX8) of *PTPRQ* in a consanguineous family with nonsyndromic hearing loss. This family came from the remote Wuling Mountains in southern China, which is a gathering place for ethnic minorities and economically suppressed, where intimate marriages often occur. Close marriages greatly increase the probability that future generations will suffer from deafness. Alleles from a common ancestor reached a homozygous state in the mother (II-3) and uncle (II-2) of the proband (III-3).

## Conclusions

In summary, we identified a novel heterozygous mutation of *SALL1* (ENST00000251020: c.1428_1429insT, p. K478QfsX38) and a new homozygous mutation of *PTPRQ* (ENST00000266688: c.1057_1057delC, p. L353SfsX8) in a Chinese family with TBS and nonsyndromic hearing loss. Our study not only confirmed the diagnosis of TBS at the molecular level but also expanded the spectrum of *SALL1* mutations and *PTPRQ* mutations. These findings may contribute to the clinical management and genetic counselling of TBS and hearing loss.

## Supplementary information

**Additional file 1**. The sequencing data of III-3.

**Additional file 2**. The sequencing data of II-3.

## Data Availability

The data used to support the findings of this study are included within the article.

## Abbreviations

SALL1: Spalt-like transcription factor 1
TBS: Townes-Brocks syndrome
CHD: Congenital heart disease
PTPRQ: Protein tyrosine phosphatase receptor type Q
PH: Pulmonary hypertension
ARM: Congenital anorectal malformations
VSD: Ventricular septal defect
